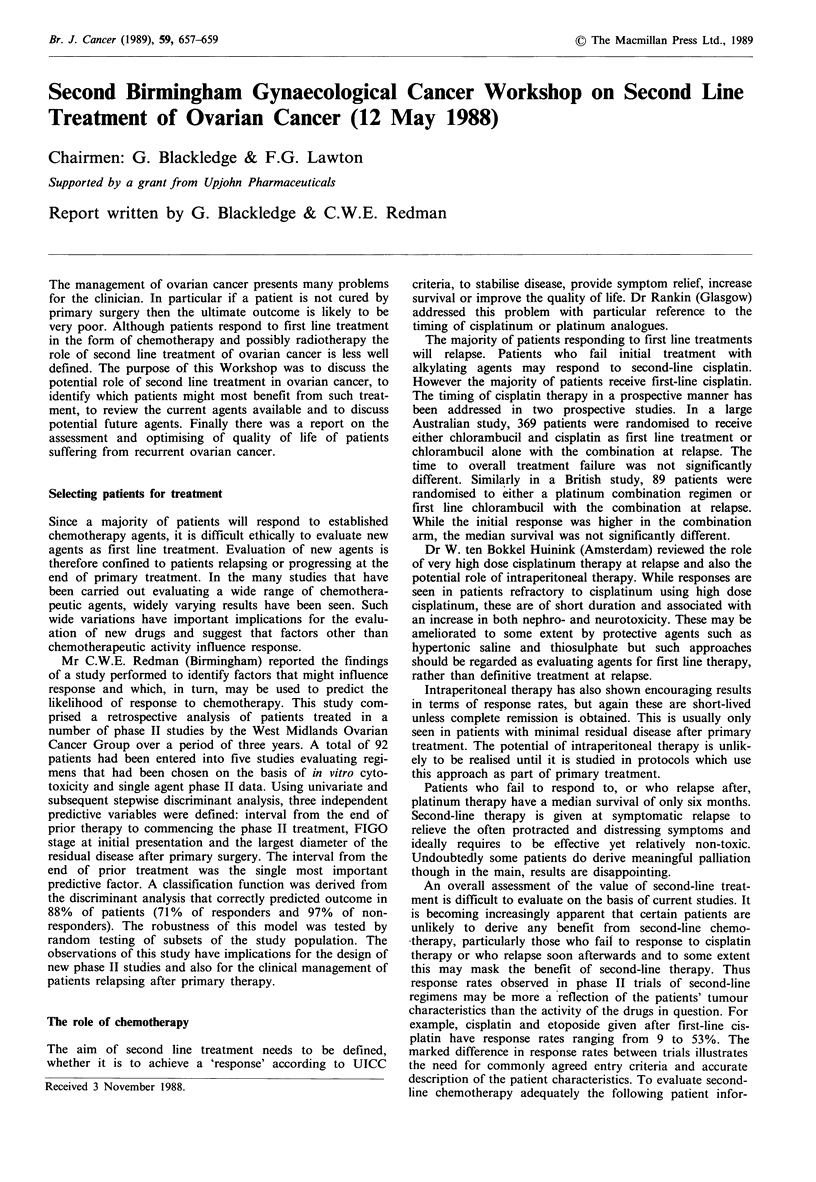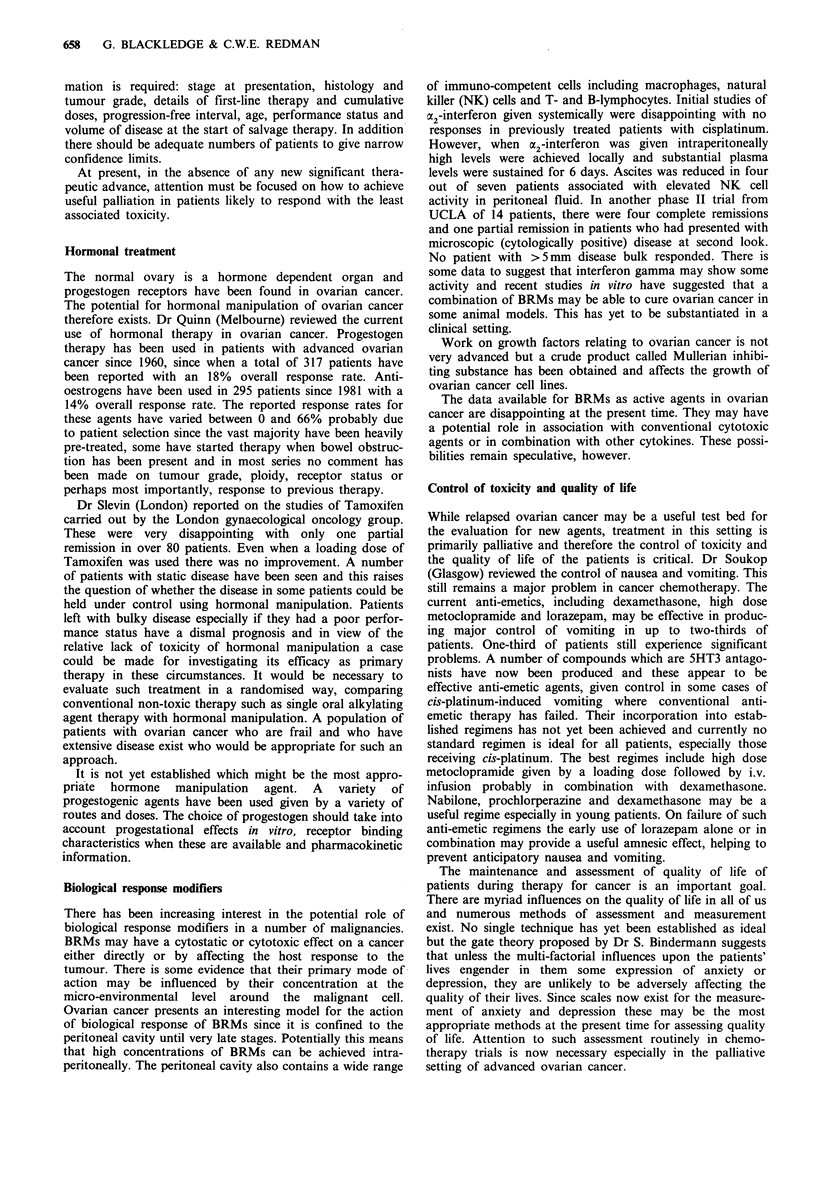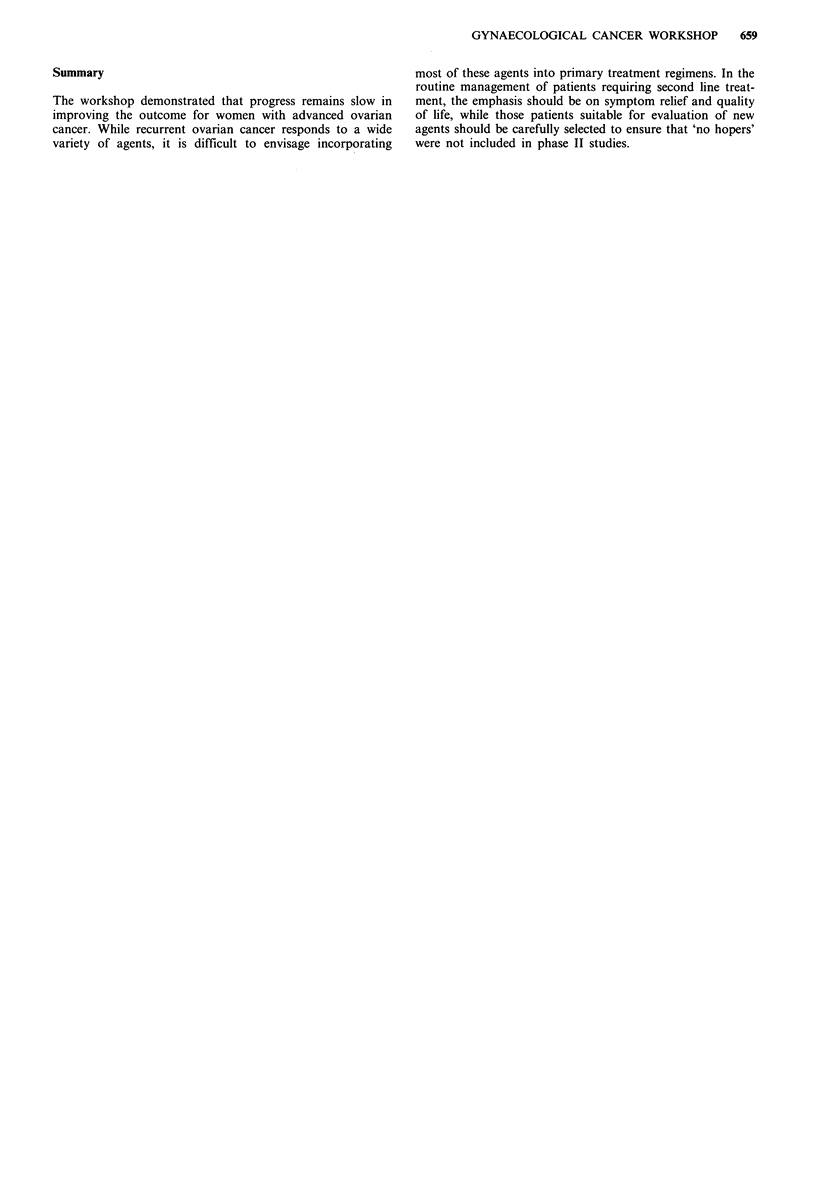# 2nd Birmingham gynaecological cancer workshop second line treatment of ovarian cancer - 12 May, 1988

**Published:** 1989-04

**Authors:** 


*Br. J. Cancer* (1989), **59**, 657–659 © The Macmillan Press Ltd., 1989

### Second Birmingham Gynaecological Cancer Workshop on Second Line Treatment of Ovarian Cancer (12 May 1988)

Chairmen: G. Blackledge & F.G. Lawton

*Supported by a grant from Upjohn Pharmaceuticals*

Report written by G. Blackledge & C.W.E. Redman

The management of ovarian cancer presents many problems for the clinician. In particular if a patient is not cured by primary surgery then the ultimate outcome is likely to be very poor. Although patients respond to first line treatment in the form of chemotherapy and possibly radiotherapy the role of second line treatment of ovarian cancer is less well defined. The purpose of this Workshop was to discuss the potential role of second line treatment in ovarian cancer, to identify which patients might most benefit from such treatment, to review the current agents available and to discuss potential future agents. Finally there was a report on the assessment and optimising of quality of life of patients suffering from recurrent ovarian cancer.

### Selecting patients for treatment

Since a majority of patients will respond to established chemotherapy agents, it is difficult ethically to evaluate new agents as first line treatment. Evaluation of new agents is therefore confined to patients relapsing or progressing at the end of primary treatment. In the many studies that have been carried out evaluating a wide range of chemotherapeutic agents, widely varying results have been seen. Such wide variations have important implications for the evaluation of new drugs and suggest that factors other than chemotherapeutic activity influence response.

Mr C.W.E. Redman (Birmingham) reported the findings of a study performed to identify factors that might influence response and which, in turn, may be used to predict the likelihood of response to chemotherapy. This study comprised a retrospective analysis of patients treated in a number of phase II studies by the West Midlands Ovarian Cancer Group over a period of three years. A total of 92 patients had been entered into five studies evaluating regimens that had been chosen on the basis of *in vitro* cytotoxicity and single agent phase II data. Using univariate and subsequent stepwise discriminant analysis, three independent predictive variables were defined: interval from the end of prior therapy to commencing the phase II treatment, FIGO stage at initial presentation and the largest diameter of the residual disease after primary surgery. The interval from the end of prior treatment was the single most important predictive factor. A classification function was derived from the discriminant analysis that correctly predicted outcome in 88% of patients (71% of responders and 97% of non-responders). The robustness of this model was tested by random testing of subsets of the study population. The observations of this study have implications for the design of new phase II studies and also for the clinical management of patients relapsing after primary therapy.

### The role of chemotherapy

The aim of second line treatment needs to be defined, whether it is to achieve a 'response' according to UICC criteria, to stabilise disease, provide symptom relief, increase survival or improve the quality of life. Dr Rankin (Glasgow) addressed this problem with particular reference to the timing of cisplatinum or platinum analogues.

The majority of patients responding to first line treatments will relapse. Patients who fail initial treatment with alkylating agents may respond to second-line cisplatin. However the majority of patients receive first-line cisplatin. The timing of cisplatin therapy in a prospective manner has been addressed in two prospective studies. In a large Australian study, 369 patients were randomised to receive either chlorambucil and cisplatin as first line treatment or chlorambucil alone with the combination at relapse. The time to overall treatment failure was not significantly different. Similarly in a British study, 89 patients were randomised to either a platinum combination regimen or first line chlorambucil with the combination at relapse. While the initial response was higher in the combination arm, the median survival was not significantly different.

Dr W. ten Bokkel Huinink (Amsterdam) reviewed the role of very high dose cisplatinum therapy at relapse and also the potential role of intraperitoneal therapy. While responses are seen in patients refractory to cisplatinum using high dose cisplatinum, these are of short duration and associated with an increase in both nephro- and neurotoxicity. These may be ameliorated to some extent by protective agents such as hypertonic saline and thiosulphate but such approaches should be regarded as evaluating agents for first line therapy, rather than definitive treatment at relapse.

Intraperitoneal therapy has also shown encouraging results in terms of response rates, but again these are short-lived unless complete remission is obtained. This is usually only seen in patients with minimal residual disease after primary treatment. The potential of intraperitoneal therapy is unlikely to be realised until it is studied in protocols which use this approach as part of primary treatment.

Patients who fail to respond to, or who relapse after, platinum therapy have a median survival of only six months. Second-line therapy is given at symptomatic relapse to relieve the often protracted and distressing symptoms and ideally requires to be effective yet relatively non-toxic. Undoubtedly some patients do derive meaningful palliation though in the main, results are disappointing.

An overall assessment of the value of second-line treatment is difficult to evaluate on the basis of current studies. It is becoming increasingly apparent that certain patients are unlikely to derive any benefit from second-line chemotherapy, particularly those who fail to response to cisplatin therapy or who relapse soon afterwards and to some extent this may mask the benefit of second-line therapy. Thus response rates observed in phase II trials of second-line regimens may be more a reflection of the patients' tumour characteristics than the activity of the drugs in question. For example, cisplatin and etoposide given after first-line cisplatin have response rates ranging from 9 to 53%. The marked difference in response rates between trials illustrates the need for commonly agreed entry criteria and accurate description of the patient characteristics. To evaluate second-line chemotherapy adequately the following patient infor-

Received 3 November 1988.

mation is required: stage at presentation, histology and tumour grade, details of first-line therapy and cumulative doses, progression-free interval, age, performance status and volume of disease at the start of salvage therapy. In addition there should be adequate numbers of patients to give narrow confidence limits.

At present, in the absence of any new significant therapeutic advance, attention must be focused on how to achieve useful palliation in patients likely to respond with the least associated toxicity.

### Hormonal treatment

The normal ovary is a hormone dependent organ and progestogen receptors have been found in ovarian cancer. The potential for hormonal manipulation of ovarian cancer therefore exists. Dr Quinn (Melbourne) reviewed the current use of hormonal therapy in ovarian cancer. Progestogen therapy has been used in patients with advanced ovarian cancer since 1960, since when a total of 317 patients have been reported with an 18% overall response rate. Anti-oestrogens have been used in 295 patients since 1981 with a 14% overall response rate. The reported response rates for these agents have varied between 0 and 66% probably due to patient selection since the vast majority have been heavily pre-treated, some have started therapy when bowel obstruction has been present and in most series no comment has been made on tumour grade, ploidy, receptor status or perhaps most importantly, response to previous therapy.

Dr Slevin (London) reported on the studies of Tamoxifen carried out by the London gynaecological oncology group. These were very disappointing with only one partial remission in over 80 patients. Even when a loading dose of Tamoxifen was used there was no improvement. A number of patients with static disease have been seen and this raises the question of whether the disease in some patients could be held under control using hormonal manipulation. Patients left with bulky disease especially if they had a poor performance status have a dismal prognosis and in view of the relative lack of toxicity of hormonal manipulation a case could be made for investigating its efficacy as primary therapy in these circumstances. It would be necessary to evaluate such treatment in a randomised way, comparing conventional non-toxic therapy such as single oral alkylating agent therapy with hormonal manipulation. A population of patients with ovarian cancer who are frail and who have extensive disease exist who would be appropriate for such an approach.

It is not yet established which might be the most appropriate hormone manipulation agent. A variety of progestogenic agents have been used given by a variety of routes and doses. The choice of progestogen should take into account progestational effects *in vitro,* receptor binding characteristics when these are available and pharmacokinetic information.

### Biological response modifiers

There has been increasing interest in the potential role of biological response modifiers in a number of malignancies. BRMs may have a cytostatic or cytotoxic effect on a cancer either directly or by affecting the host response to the tumour. There is some evidence that their primary mode of action may be influenced by their concentration at the micro-environmental level around the malignant cell. Ovarian cancer presents an interesting model for the action of biological response of BRMs since it is confined to the peritoneal cavity until very late stages. Potentially this means that high concentrations of BRMs can be achieved intraperitoneally. The peritoneal cavity also contains a wide range of immuno-competent cells including macrophages, natural killer (NK) cells and T- and B-lymphocytes. Initial studies of $\alpha_2$-interferon given systemically were disappointing with no responses in previously treated patients with cisplatinum. However, when $\alpha_2$-interferon was given intraperitoneally high levels were achieved locally and substantial plasma levels were sustained for 6 days. Ascites was reduced in four out of seven patients associated with elevated NK cell activity in peritoneal fluid. In another phase II trial from UCLA of 14 patients, there were four complete remissions and one partial remission in patients who had presented with microscopic (cytologically positive) disease at second look. No patient with $>5$ mm disease bulk responded. There is some data to suggest that interferon gamma may show some activity and recent studies *in vitro* have suggested that a combination of BRMs may be able to cure ovarian cancer in some animal models. This has yet to be substantiated in a clinical setting.

Work on growth factors relating to ovarian cancer is not very advanced but a crude product called Mullerian inhibiting substance has been obtained and affects the growth of ovarian cancer cell lines.

The data available for BRMs as active agents in ovarian cancer are disappointing at the present time. They may have a potential role in association with conventional cytotoxic agents or in combination with other cytokines. These possibilities remain speculative, however.

### Control of toxicity and quality of life

While relapsed ovarian cancer may be a useful test bed for the evaluation for new agents, treatment in this setting is primarily palliative and therefore the control of toxicity and the quality of life of the patients is critical. Dr Soukop (Glasgow) reviewed the control of nausea and vomiting. This still remains a major problem in cancer chemotherapy. The current anti-emetics, including dexamethasone, high dose metoclopramide and lorazepam, may be effective in producing major control of vomiting in up to two-thirds of patients. One-third of patients still experience significant problems. A number of compounds which are 5HT3 antagonists have now been produced and these appear to be effective anti-emetic agents, given control in some cases of *cis*-platinum-induced vomiting where conventional anti-emetic therapy has failed. Their incorporation into established regimens has not yet been achieved and currently no standard regimen is ideal for all patients, especially those receiving *cis*-platinum. The best regimes include high dose metoclopramide given by a loading dose followed by i.v. infusion probably in combination with dexamethasone. Nabilone, prochlorperazine and dexamethasone may be a useful regime especially in young patients. On failure of such anti-emetic regimens the early use of lorazepam alone or in combination may provide a useful amnesic effect, helping to prevent anticipatory nausea and vomiting.

The maintenance and assessment of quality of life of patients during therapy for cancer is an important goal. There are myriad influences on the quality of life in all of us and numerous methods of assessment and measurement exist. No single technique has yet been established as ideal but the gate theory proposed by Dr S. Bindermann suggests that unless the multi-factorial influences upon the patients' lives engender in them some expression of anxiety or depression, they are unlikely to be adversely affecting the quality of their lives. Since scales now exist for the measurement of anxiety and depression these may be the most appropriate methods at the present time for assessing quality of life. Attention to such assessment routinely in chemotherapy trials is now necessary especially in the palliative setting of advanced ovarian cancer.

**Summary**

The workshop demonstrated that progress remains slow in improving the outcome for women with advanced ovarian cancer. While recurrent ovarian cancer responds to a wide variety of agents, it is difficult to envisage incorporating most of these agents into primary treatment regimens. In the routine management of patients requiring second line treatment, the emphasis should be on symptom relief and quality of life, while those patients suitable for evaluation of new agents should be carefully selected to ensure that 'no hopers' were not included in phase II studies.